# Ovarian gonadoblastoma with dysgerminoma in a girl with 46,XX karyotype 17a-hydroxylase/17, 20-lyase deficiency: A case report and literature review

**DOI:** 10.3389/fendo.2022.989695

**Published:** 2022-12-15

**Authors:** Min Yin, Jiaxin Yang, Qinjie Tian, Xinyue Zhang

**Affiliations:** Department of Obstetrics and Gynecology, National Clinical Research Center for Obstetric and Gynecologic Diseases, Peking Union Medical College Hospital, Chinese Academy of Medical Sciences and Peking Union Medical College, Beijing, China

**Keywords:** congenital adrenal hyperplasia, *CYP17A1* gene, ovarian gonadoblastoma, dysgerminoma, 17α-hydroxylase/17,20-lyase deficiency

## Abstract

17α−hydroxylase/17,20−lyase deficiency (17-OHD), caused by mutations in the gene of the cytochrome P450 family 17 subfamily A member 1 (CYP17A1), is a rare type of congenital adrenal hyperplasia (CAH), usually characterized by cortisol and sex steroid deficiency combined with excessive mineralocorticoid. Gonadoblastoma is a relatively rare ovarian tumor that is frequently seen among patients with 46,XY gonadal dysgenesis. Rarely have they been reported in female patients with normal 46,XX karyotype. Here, we report an interesting case of an 11-year-old Chinese girl who presented acute abdominal pain that was later attributed to tumor rupture of right ovarian gonadoblastoma with dysgerminoma. Further evaluations revealed hypertension and hypokalemia. Hormonal findings showed increased progesterone, hypergonadotropic hypogonadism, and low cortisol levels. Her chromosome karyotype was 46,XX without Y chromosome material detected. Genetic analysis revealed that the patient had a homozygous pathogenic variant c.985_987delTACinsAA (p.Y329Kfs*90) in exon 6 of the *CYP17A1* gene and that her parents were all heterozygous carriers of this pathogenic variant. Due to the variable clinical manifestations of 17-OHD, meticulous assessment including genetic analysis is necessary. Further study is warranted to unravel the mechanism of gonadoblastoma in a patient with normal karyotypes.

## Introduction

Congenital adrenal hyperplasia (CAH) refers to a group of syndromes caused by inherited deficiencies in one of five enzymes involved in the biosynthesis of cortisol from cholesterol (1). The majority of CAH cases (90–95%) are caused by 21α-hydroxylase deficiency, whereas 17α-hydroxylase/17,20-lyase deficiency (17-OHD) is the least frequent form of the condition and only accounts for 1% of all CAH cases (2). The cytochrome P450c17 enzyme catalyzes both 17α-hydroxylase activity and 17,20-lyase activity, which play a pivotal role in the production of cortisol and sex hormones. This enzyme is encoded by the *CYP17A1* gene, located on chromosome 10q24.3. Mutations in the *CYP17A1* gene lead to a deficiency in 17α-hydroxylase/17,20-lyase. Due to blockage of sex hormone synthesis, 17-OHD causes differences/disorder of sex development (DSD) in 46,XY, while sexual infantilism and primary amenorrhea in 46,XX. Moreover, insufficient glucocorticoids in turn increase adrenocorticotropic hormone (ACTH) secretion, leading to adrenal hyperplasia and excessive mineralocorticoid, causing hypertension and hypokalemia (3).

Gonadoblastomas are relatively rare ovarian tumors consisting of sex cord and primitive germ cell components (4). Although the great majority of gonadoblastomas occur in individuals with 46,XY gonadal dysgenesis, a substantial number arise in individuals with normal 46,XX karyotype (5). In this study, we reported a case of an 11-year-old girl with 46,XX karyotype who initially presented with ovarian gonadoblastoma with dysgerminoma and was finally diagnosed with 17-OHD caused by p.Y329Kfs*90 homozygous pathogenic variant.

## Case report

**Figure 1:**
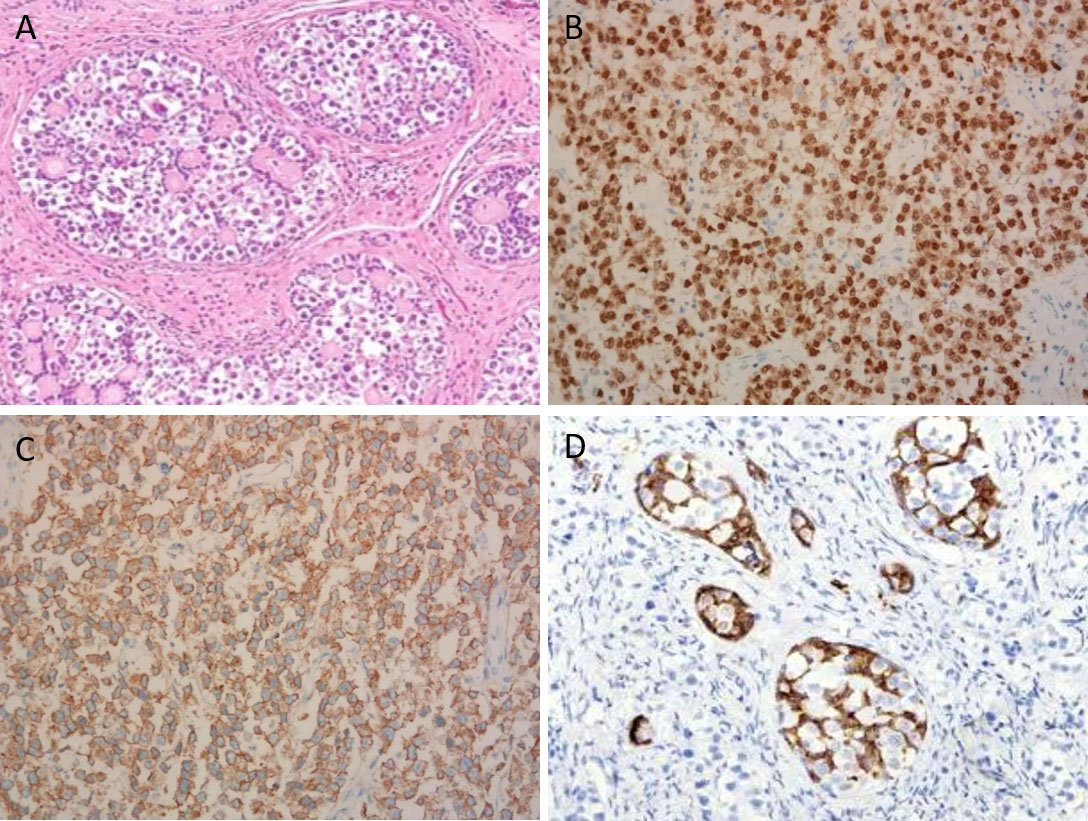
Microscopic view of the ovarian gonadoblastoma with coexisting dysgerminoma (×200). **(A)** A mixture of germ cells, elements of the sex cord, and hyaline bodies were placed in large nests and lobes. **(B)** Dysgerminoma component demonstrating positive nuclear staining for OCT4. **(C)** Dysgerminoma component demonstrating positive membranous staining for CD117. **(D)** Sex cord component demonstrating positive cytoplasmic staining for α-inhibin.

An 11-year-old girl was admitted to the local hospital for abdominal pain in April 2022. CT revealed a large pelvic mass measuring 10 × 8 cm in diameter, with suspicion of adnexal torsion or tumor rupture. Serum tumor markers, including carbohydrate antigen (CA) 125, CA199, carcinogenic embryonic antigen (CEA), alpha-fetoprotein (AFP) and β-human chorionic gonadotropin (β-HCG) were in the normal range. An emergent laparoscopy showed a ruptured ovarian tumor measuring 10 × 8 × 6 cm on the right side, along with approximately 50 ml of intra-abdominal bleeding. The root of the right adnexa was twisted 720 degrees. The uterus and the left ovary were small in size, and no enlarged retroperitoneal lymph nodes were noted. Right salpingo-oophorectomy was performed. The sample was placed in an endo bag and exteriorized through the incision. Microscopically, a mixture of germ cells, sex cord elements, and hyaline bodies were arranged in large nests and lobules. The sex cord cells were arranged at the periphery of these nests (**Figure 1A**). The dysgerminoma component showed positive immunohistochemical staining (IHC) for OCT4 (**Figure 1B**), SALL4 and CD117 (**Figure 1C**). An α-inhibin IHC staining showed positivity in the component of the sex cord of the nests (**Figure 1D**). Other IHC markers, including estrogen receptor (ER), progesterone receptor (PR), AFP, CEA, β-HCG, testis-specific protein Y-encoded (TSPY), and epithelial membrane antigen (EMA), were negative. Based on these findings, the diagnosis of “gonadoblastoma with dysgerminoma” was made.

The patient was then referred to our hospital’s outpatient in May 2022 for further evaluation and treatment. Menstruation has not yet begun. Physical examination showed that her blood pressure was 148/106 mmHg, her body weight was 40 kg, and her height was 160 cm. Her breasts were in Tanner stage I, without pubic or axillary hair. There was no clitoromegaly and the external genitalia were phenotypically female and infantile. Blood biochemistry tests showed hypokalemia and normal plasma sodium and chlorine level. The following hormonal tests revealed low estradiol (E2) and testosterone (T), along with increased levels of progesterone (P), follicle stimulating hormone (FSH) and luteinizing hormone (LH). Furthermore, decreased cortisol and elevated ACTH levels were observed (**Table 1**). Serum tumor markers were still within the normal range.

**Table 1:** Hormone levels and biochemical parameters of the patient.

Items	Results	Reference range
E2 (ng/mL)	<0.02 ↓	Follicular phase, 0.039-0.375
T (ng/mL)	<0.02 ↓	<0.41
P (ng/mL)	5.83 ↑	Follicular phase, <0.31
FSH (IU/L)	105.92 ↑	Follicular phase, <10.00
LH (IU/L)	34.25 ↑	Follicular phase, 2.12-10.89
PRL (ng/mL)	13.20	<30.0
Pregnenolone (ng/mL)	3.51 ↑	<2.30
11-Deoxycorticosterone (ng/mL)	1.322 ↑	<0.300
Corticosterone (ng/mL)	116.60 ↑	0.18-19.70
ACTH (pg/mL)	68.20 ↑	7.20-63.30
Cortisol-8am (ng/mL)	<0.40 ↓	48.2-195
Aldosterone (ng/mL)	<0.02	<0.22
Potassium (mmol/L)	3.12 ↓	3.5-5.5
Sodium (mmol/L)	142	135-145
Chlorine (mmol/L)	107	96-111

E2, estradiol; T, testosterone; P, progesterone; FSH, follicle-stimulating hormone; LH, luteinizing hormone; PRL, prolactin; ACTH, adrenocorticotropic hormone. The symbols ↑ means above normal, the symbols ↓ means below normal.

**Figure 2:**
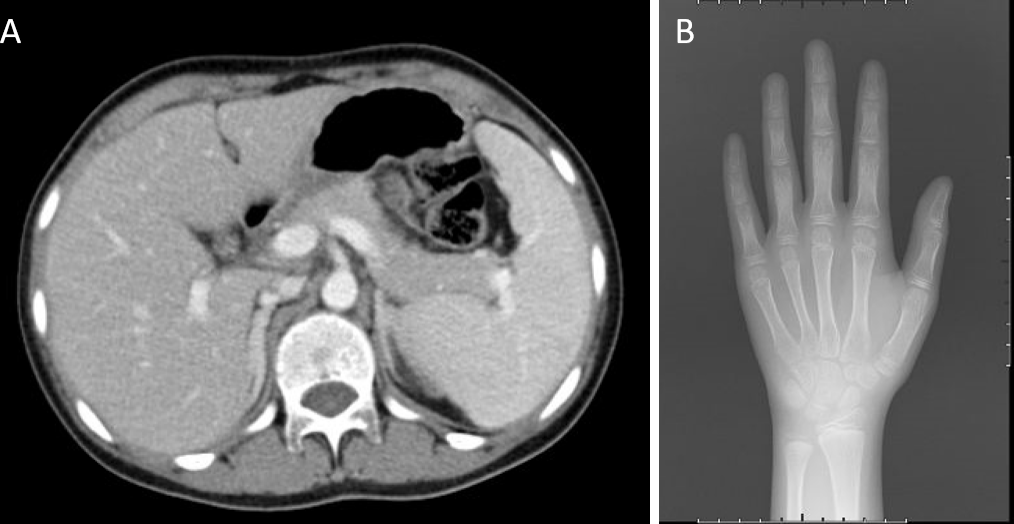
Imaging examinations. **(A)** An abdominal CT scan revealed no apparent hyperplasia in the bilateral adrenal gland. **(B)** X-ray showed her bone age was 8 years old.

**Figure 3:**
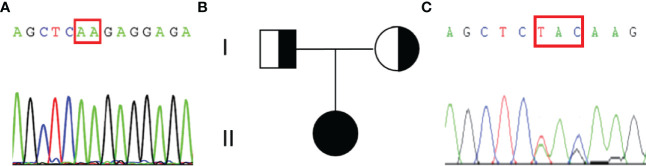
Results of the sequencing of CYP17A1 gene and the pedigree of the patient’s family. **(A)**The patient was homozygous for the pathogenic variant c.985_987delTACinsAA (p.Y329Kfs*90). The red rectangular represents the altered nuclear acid. **(B)** Pedigree of the patient’s family. **(C)** Her parents were all heterozygous for c.985_987delTACinsAA (p.Y329Kfs*90) pathogenic variant.

An abdominal CT scan revealed no apparent hyperplasia in the bilateral adrenal gland (**Figure 2A**). Based on the X-ray, her bone age was 3 years younger than her actual age (**Figure 2B**). Karyotype analysis from the peripheral blood sample revealed a normal female 46,XX pattern and ruled out the possibility of mosaicism. To further exclude the possibility of a low level of Y chromosome material, fluorescence *in situ* hybridization (FISH) analysis using a probe specific for the *sex-determining region of Y-chromosome (SRY)* gene and *zinc-finger Y (ZFY)* gene locus at the Y chromosome was performed and no signal was detected for the *SRY* and *ZFY* locus. To exclude the possibility of somatic mosaicism, additional molecular analyzes were conducted in both the gonadal stroma and tumor tissue. Quantitative fluorescence-polymerase chain reaction (QF-PCR) results showed no amplification of the *SRY* in both the gonadal stroma and tumor tissue. Further whole-exome sequencing of the patient indicated that she was homozygous for c.985_987delTACinsAA (p.Y329Kfs*90) in exon 6 of the *CYP17A1* gene (**Figure 3A**). No other genetic variant in gonadal development was found. She has no siblings and peripheral blood samples from her parents were also sent to analyze the genetic sequence of CYP17A1. Although her parents were apparently not consanguineous, they were all heterozygous carriers of this pathogenic variant (**Figures 3B, C**).

As no Y-chromosome material was identified in peripheral blood, gonadal stroma, and tumor, the risk of malignancy of the left ovary was estimated to be low. Therefore, the contralateral ovary was preserved. According to the International Federation of Gynecology and Obstetrics (FIGO) staging classification for cancer of the ovary, the pathological staging was stage I gonadoblastoma with dysgerminoma, and no adjuvant chemotherapy was needed based on the National Comprehensive Cancer Network (NCCN) guidelines. Glucocorticoid supplementary treatment was initiated. The patient was prescribed prednisone starting at 5 mg/day. Three months later, her blood pressure varied between 117-130/72-85 mmHg, and plasma levels of potassium, cortisol, and ACTH returned to the normal range. After communicating with the patient and her parents about her condition, supplementary treatment with sex hormones will be used later to promote the development of secondary sexual characteristics and induce an artificial menstrual cycle. During the follow-up period, physical examination, blood parameters, including sex hormone, cortisol, ACTH, aldosterone, blood electrolyte levels, and pelvic ultrasound will be regularly monitored. Currently, the follow-up is still ongoing.

## Discussion

17-OHD is a rare kind of congenital adrenal hyperplasia (CAH) characterized by the failure to synthesize cortisol, adrenal androgens, and gonadal steroids (6). Defects in 17α-hydroxylase/17,20-lyase activities result in low cortisol levels and a reduction in dehydroepiandrosterone (DHEA) and androstenedione, which in turn results in low estradiol and testosterone levels (7). Negative feedback is diminished by lower levels of cortisol, and consequentially induces ACTH overproduction, adrenal hyperplasia, and excessive synthesis of mineralocorticoid precursors, such as 11-deoxycorticosterone and corticosterone, resulting in low-renin hypertension and hypokalaemia. A deficiency of sex hormones causes primary amenorrhea in females, and 46,XY DSD in males, which manifests with small testicles, small penis, and mammary gland development.

Biglieri et al. described the first 17-OHD case in 1966, which involved a 35-year-old woman with hypertension and delayed menstruation as the primary symptoms (8). To date, more than 100 pathogenic variants have been reported in the *CYP17A1* gene, and most are associated with a classic phenotype of combined 17α-hydroxylase/17,20-lyase deficiency. A smaller number of *CYP17A1* missense variants have been reported to exhibit partial impairment of 17α-hydroxylase/17,20-lyase activity. Based on the degree of enzyme deficiency, 17-OHD is classified chiefly into complete and partial type deficiency. Specific *CYP17A1* gene variants have been reported to cause a partial loss of 17a-hydroxylase/17,20-lyase activities or dissociation of the 17a-hydroxylase and 17,20-lyase functions (9). Hypergonadotropic hypogonadism and high serum levels of ACTH and mineralocorticoids are found in complete and partial 17-OHD patients. Patients with partial 17-OHD have different clinical characteristics such as the development of breasts and pubic hair, and oligomenorrhea or secondary amenorrhea, due to the various degrees of estrogenic and androgenic impacts (10).

Until now, numerous pathogenic variants in the *CYP17A1* gene have been identified in 17-OHD patients, and differ among racial and geographic areas. Tyrosine at amino acid position 329 is particularly critical for the appropriate functioning of the P450c17 enzyme (11). Tyrosine (Y)-329-Lysine (K) is the first impacted amino acid due to a frameshift in exon 6 of the *CYP17A1* gene, which occurs when the nucleotide 985 to 987 (TAC) was changed by AA. This frameshift pathogenic variant finally leads to the premature stop codon of 418TGA and generates a truncated protein containing only 417 amino acids without the heme-binding region of codons 435 and 455, which plays a key role in the catalytic function of the enzyme. Therefore, the loss of the pivotal functional domain results in the complete loss of 17-hydroxylase/17,20-lyase activity.

In 2003, a Korean compound heterozygote patient with primary amenorrhea, hypokalemia, and hypertension, was first diagnosed with this pathogenic variant (12). Subsequently, this pathogenic variant was found in many Chinese 17-OHD patients. Tian et al. reported cases with the pathogenic variant p.Y329Kfs*90 and assumed that this was common in Asian people (13). Another study reported that 10 out of 15 (66.7%) Chinese 17α-OHD patients carried the pathogenic variant p.Y329Kfs*90 (14). To clarify the prevalence of 17-OHD pathogenic variants in China, Wang et al. (15) searched PubMed for all published English papers about 17-OHD and summarized all reported *CYP17A1* gene pathogenic variants. In total, genetic variants were reported in 181 Chinese cases; of them, 70 (38.6%) had a pathogenic variant p.Y329Kfs*90.

Gonadoblastoma, an uncommon ovarian tumor, occurs almost always in patients with dysgenetic gonads associated with DSD and an aberrant karyotype (4). Although gonadoblastomas are benign themselves, they are frequently associated with invasive germ-cell malignant tumors. The most common malignancy is pure dysgerminoma, whereas other variants include immature teratoma, embryonal carcinoma, yolk sac tumor, and choriocarcinoma (16). In 1970, Scully reviewed 74 cases of ovarian gonadoblastoma and more than 90% of the reviewed cases had a Y chromosome. As a result, the Y chromosome is believed to be the locus of an oncogene necessary for the development of a gonadoblastoma (17). Subsequently, it was postulated that GBY (gonadoblastoma locus on the Y chromosome) exerts oncogenic effects in dysgenetic gonads and the *TSPY* gene was established as the putative gene for GBY (18). Y chromosome increases the risk of gonadal malignancy by 15% to 50% in patients who have partial or total gonadal dysgenesis, therefore, preventive bilateral gonadectomy is usually recommended (19). Due to the young age at diagnosis, it is crucial to rule out the presence of Y chromosomal material. Typically, peripheral blood lymphocyte karyotyping is performed to check for Y chromosomes in the germline; however, a small chromosomal fragment containing Y chromosome material may be missed (20). The unique approach of incorporating molecular techniques, such as FISH and QF-PCR, were used to confirm the absence of Y chromosome material in peripheral blood and tumor tissue to exclude gonadal mosaicism or the presence of a fragment of the Y chromosome too small to be detected using routine cytogenetic analysis, therefore accurately determining risks to the contralateral gonad (21).

Although most cases of gonadoblastoma occur in sexually abnormal patients with a Y chromosome, a substantial number of cases arise in females with a normal 46,XX karyotype. We reviewed the literature since 1990 and identified 13 female cases of gonadoblastoma with a normal 46,XX karyotype (22–34). **Table 2** summarized the clinical characteristics of the 13 cases that we found in the literature. The age at diagnosis varied between 9 to 34 years old. Of the 14 cases, 8 (57%) occurred in women older than 18 years, 4 of whom were fertile. Most (86%) gonadoblastomas were unilateral. Preoperatively, most of them reported abnormally elevated serum tumor markers, such as β-HCG, AFP, and lactic dehydrogenase (LDH). Only one patient reported elevated levels of testosterone, while others did not report hormone levels. Kanaga et al. (29) reported a 14-year-old girl who presented with abdominal distension, excessive hair growth over the body, and hoarseness of voice. An elevation of LDH, β-HCG, and testosterone levels suggested the probable diagnosis of germ cell tumor, mostly dysgerminoma. Laparotomy revealed a large left ovarian gonadoblastoma with dysgerminoma. After surgery and combination chemotherapy, the levels of β-HCG, LDH, and testosterone were reduced to normal. Patients with gonadoblastoma and dysgerminoma generally have an excellent prognosis. When gonadoblastoma is accompanied by more aggressive germ cell tumors, such as yolk sac tumor, embryonal carcinoma, immature teratoma, and choriocarcinoma, the prognosis is different. Even though no hypothesis for the occurrence of these neoplasms has yet been put out in the literature, several pathological studies suggested that they likely developed *via* a completely distinct molecular pathway from those that arose in people who have a DSD (4, 5).

**Table 2:** Summary of published female cases of gonadoblastoma with normal 46, XX karyotype.

No.	Author	Year	Age at diagnosis	Clinical presentation	Fertile status	Hormonal abnormalities	Laterality	Surgical procedure	Pathological findings in addition to gonadoblastoma	Adjuvant therapy
1	Erhan Y et al (22)	1992	26	Abdominal mass during pregnancy	Fertile	NR	Right	TAH + BSO	Dysgerminoma	Chemotherapy
2	Obata NH et al (23)	1995	10	Abdominal discomfort	No	NR	Bilateral	USO + right ovarian cystectomy	Left with dysgerminoma, right with dysgerminoma and yolk sac tumor	Chemotherapy
3	Zhao S et al (24)	2000	27	Abdominal pain	Fertile	NR	Right	USO + chemotherapy + later TAH + USO + LND + omentectomy	Choriocarcinoma, embryonal carcinoma, yolk sac tumor, immature teratoma and dysgerminoma	Chemotherapy
4	Erdemoglu E et al (25)	2007	19	Abdominal mass and pain	No	NR	Unilateral	USO	Endodermal sinus tumor	None
5	Yilmaz B et al (26)	2010	20	Abdominal mass	No	NR	Bilateral	BSO	Bilateral with dysgerminoma	Radiation and chemotherapy
6	Koo YJ et al (27)	2011	34	Vaginal bleeding	Fertile	NR	Left	USO + para-aortic LND	Dysgerminoma	Chemotherapy
7	Esin S et al (28)	2011	15	Vaginal bleeding	No	NR	Right	USO	Dysgerminoma	None
8	Kanagal DV et al (29)	2013	14	Abdominal distension	No	Elevated testosterone level	Left	USO + pelvic and para-aortic LND + infra-colic omentectomy	Dysgerminoma	Chemotherapy
9	Kulkarni MM et al (30)	2016	20	Abdominal pain	Fertile	NR	Left	USO + omental biopsy	Dysgerminoma	None
10	McCuaig JM et al (31)	2017	20	Oligomenorrhea and menorrhagia	No	NR	Left	USO	Dysgerminoma	None
11	Roth LM et al (32)	2019	9	Abdominal pain and a right adnexal mass	No	NR	Right	USO	Mixed germ cell tumor.	Chemotherapy
12	Raafey MA et al (33)	2020	10	Abdominal pain, abdominal distention and fever	No	NR	Left	USO	Dysgerminoma	Chemotherapy
13	Chandrapattan P et al (34)	2022	9	Signs of virilization and contrasexual pubertal development	No	NR	Right	USO + pelvic LND + infra-colic omentectomy	Dysgerminoma	Chemotherapy

BSO, bilateral salpingo-oophorectomy; USO, unilateral salpingo-oophorectomy; LND, lymph node dissection; TAH, total abdominal hysterectomy; NR, not reported.

At present, there is limited evidence on the gonadal-malignancy risk of patients with 17-OHD, and there is no clear guidance regarding prophylactic gonadectomies. According to our experience, of the 20 female 17-OHD patients with 46,XY, two (10%) patients had gonadal tumors, one with a Leydig cell tumor and the other with a Sertoli cell tumor (35). The gonads of these two patients were located in the abdomen. Genetic analysis of the *CYP17A1* gene showed that one patient had a homozygous pathogenic variant p.T390R in exon 7 of *CYP17A1*, and another had a homozygous pathogenic variant p.Y329Kfs*90, whose variant type was the same as the case we reported here. Given the fact that the malignancy rate for these patients is quite high, 17-OHD patients with Y chromosome material are recommended to undergo prophylactic gonadectomy to prevent gonadal malignancy. In our case, no Y chromosome material was identified in peripheral blood, gonadal stroma, and tumor, and the risk of malignancy of the contralateral ovary was estimated to be low. So, the second surgery for contralateral ovary resection was avoided.

In the management of 17-OHD, glucocorticoid administration is the key therapy to suppress adrenal hyperplasia and normalize blood pressure as well as plasma potassium level. Treatment should be individualized and the dose should be adjusted according to the patient’s blood pressure, plasma potassium, and hormone levels (36). In cases where glucocorticoids alone cannot control blood pressure and potassium levels well, mineralocorticoid receptor antagonists could be added. Supplementation therapy of sex steroid hormones should be initiated in adolescent female patients to promote the development of secondary sex characteristics. Additionally, cyclical estrogen and progestin therapy are required in patients with an intact uterus to induce menstruation (37).

## Conclusion

In summary, 17-OHD is uncommon and challenging in clinical practice. We describe the extremely rare case of a 17-OHD female patient with normal 46,XX karyotype accompanied by ovarian gonadoblastoma with dysgerminoma. Due to the variable clinical characteristics of 17-OHD patients, a meticulous assessment is necessary, including genetic analysis. Further study is warranted to unravel the mechanism of gonadoblastoma in patient with normal karyotypes.

## Data availability statement

The original contributions presented in the study are included in the article/supplementary material. Further inquiries can be directed to the corresponding author.

## Ethics statement

The studies involving human participants were reviewed and approved by Peking Union Medical College Hospital. Written informed consent to participate in this study was provided by the participants’ legal guardian/next of kin.

## Author contributions

MY: Writing and literature search. JY and QT: Concept, design, and medical practice. XZ: Analysis and interpretation. All authors contributed to the article and approved the submitted version.
